# HECT domain interaction with ubiquitin binding sites on Tsg101-UEV controls HIV-1 egress, maturation, and infectivity

**DOI:** 10.1016/j.jbc.2023.102901

**Published:** 2023-01-13

**Authors:** David A. Nyenhuis, Rohith Rajasekaran, Susan Watanabe, Marie-Paule Strub, Mahfuz Khan, Michael Powell, Carol A. Carter, Nico Tjandra

**Affiliations:** 1 Biochemistry and Biophysics Center, National Heart, Lung, and Blood Institute, National Institutes of Health, Bethesda, Maryland, USA; 2 Department of Microbiology & Immunology, Renaissance School of Medicine, Stony Brook University, Stony Brook, New York, USA; 3 Department of Microbiology & Immunology, Morehouse School of Medicine, Atlanta, Georgia, USA

**Keywords:** Nedd4, ubiquitin, Tsg101, HECT, UEV, NMR, viral budding, endocytic sorting, HECT, homologous to E6AP carboxyl terminus, Nedd4-1, neural precursor cell-expressed developmentally downregulated 4-1, Tsg101, tumor susceptibility gene 101, Ub, ubiquitin, RING, really interesting new gene, ESCRT, endosomal sorting complex required for transport, DOTA, 1,4,7,10-tetraazacyclododecane-1,4,7,10-tetraacetic acid, PRE, paramagnetic relaxation enhancement, PCS, pseudocontact shift, UEV, ubiquitin E2 variant

## Abstract

The HECT domain of HECT E3 ligases consists of flexibly linked N- and C-terminal lobes, with a ubiquitin (Ub) donor site on the C-lobe that is directly involved in substrate modification. HECT ligases also possess a secondary Ub binding site in the N-lobe, which is thought to play a role in processivity, specificity, or regulation. Here, we report the use of paramagnetic solution NMR to characterize a complex formed between the isolated HECT domain of neural precursor cell-expressed developmentally downregulated 4-1 and the ubiquitin E2 variant (UEV) domain of tumor susceptibility gene 101 (Tsg101). Both proteins are involved in endosomal trafficking, a process driven by Ub signaling, and are hijacked by viral pathogens for particle assembly; however, a direct interaction between them has not been described, and the mechanism by which the HECT E3 ligase contributes to pathogen formation has not been elucidated. We provide evidence for their association, consisting of multiple sites on the neural precursor cell-expressed developmentally downregulated 4-1 HECT domain and elements of the Tsg101 UEV domain involved in noncovalent ubiquitin binding. Furthermore, we show using an established reporter assay that HECT residues perturbed by UEV proximity define determinants of viral maturation and infectivity. These results suggest the UEV interaction is a determinant of HECT activity in Ub signaling. As the endosomal trafficking pathway is hijacked by several human pathogens for egress, the HECT-UEV interaction could represent a potential novel target for therapeutic intervention.

Ubiquitin (Ub) is a small (8.6 kDa) regulatory protein found in eukaryotic cells that is employed for signaling an array of cellular processes, including protein targeting for degradation, stress responses, DNA repair, and cell cycle progression (1, 2). Signal diversity is achieved by linkage of Ub to sites on the target protein through one or more lysine residues on the Ub, permitting monoubiquitylation, multi-monoubiquitylation, or polyubiquitylation in straight or branching chains (2). Within the chain, the carboxy terminus of one Ub monomer is linked to the ε-amino group of a lysine residue or to the α-amino group of the N-terminal methionine of another Ub monomer *via* an isopeptide bond. The general process of Ub substrate modification involves a coordinated sequential action between E1 Ub-activating, E2 Ub conjugating, and E3 Ub ligating enzymes, where Ub is activated *via* thioester bond formation before being transferred to an E2 enzyme *via* a transthiolation reaction. The mechanism of Ub transfer to the target protein then differs depending on the type of E3 ligase which fall generally into the really interesting new gene (RING) monomer or dimer, RING between RING, and homologous to E6AP carboxyl terminus (HECT) classes (3).

The RING and RING between RING E3s serve as a template for the assembly of an E3-E2-Ub-substrate complex, from which nucleophilic attack by a lysine residue on the substrate displaces the Ub from the E2 to form the final substrate-Ub isopeptide bond (3, 4). Further, it has been shown structurally and *via* single molecule Förster resonance energy transfer that this complex also involves additional scaffolding from an accessory ubiquitin E2 variant (UEV) domain (4, 5). Such domains are structurally homologous to E2 enzymes but lack the catalytic cysteine that supports covalent Ub linkage. The HECT domain of HECT E3 ligases, by contrast, consists of flexibly linked N- and C-terminal lobes, with an initial transthiolation of Ub from the E2: Ub conjugate to a donor site on the HECT C-lobe, after which the E2 is no longer required, and the HECT E3 ligase completes the transfer of Ub to the substrate (3). HECT ligases also possess a secondary Ub binding site in the N-lobe, which is thought to play a role in processivity, target selection, and regulation (6).

Here, we report the formation of an encounter-like complex between the HECT domain of the E3 ligase neural precursor cell-expressed developmentally downregulated 4-1 (Nedd4-1) and the UEV domain of the Tsg101 protein. The latter is a component of the endosomal sorting complex required for transport-I (ESCRT-I), one of four (ESCRT-0, -I, -II, -III) complexes that comprise the endosomal trafficking machinery, in which Nedd4-1 or one of its isoforms functions in protein ubiquitylation (7). Tsg101/ESCRT machinery is hijacked for budding of several enveloped viruses [reviewed in (8)]. Interestingly, for some of these viruses, including Ebola virus and the human T-cell leukemia virus type1, both Nedd4 and Tsg101 bind directly to viral-encoded proteins to promote egress (7, 9), while for others, only one of the proteins is known to bind directly. For example, in the case of HIV-1, Tsg101 but not Nedd4 binds the viral structural protein Gag (10), yet Nedd4 function is still strongly implicated in budding (11, 12, 13). The avian sarcoma-leukemia virus gag binds only Nedd4 directly (14), but Tsg101 function is again implicated in budding (15, 16). Indeed, the Nedd4-1 isomer Nedd4L (Nedd4-2) and the related Nedd4-2s, which binds HIV-1 gag directly, can rescue HIV-1 production when direct Tsg101 binding to Gag is impaired (17, 18, 19). Even so, rescue requires expression of the Tsg101 protein.

Taking advantage of the increased sensitivity afforded by lanthanide tags, we used solution NMR to test for UEVHECT interaction and revealed the existence of an interaction between these domains. Our finding is consistent with the suggestion that the HECT domain of the yeast Nedd4 ortholog Rsp5 interacts *in trans* with the UEV domain of Vps23 (20), the yeast Tsg101 ortholog. The work described here represents the first structural characterization of an interaction between HECT and UEV elements.

The HECT–UEV interaction was too weak to be mapped by NMR chemical shift perturbation. Instead, we used previously characterized lanthanide complexed DOTA (1,4,7,10-tetraazacyclododecane-1,4,7,10-tetraacetic acid) tags, bound to several sites on the HECT domain, to determine intermolecular paramagnetic relaxation enhancement (PRE) and pseudocontact shifts (PCSs) on the ^1^H nuclei in the Tsg101 UEV domain (21). Similar to previously identified weak interactions characterized by paramagnetic solution NMR, the complex is encounter-like with weak orientational preference. Further, it involves the Tsg101 UEV domain binding in an interlobe region near both the canonical E2 site and the noncovalent exosite on one surface of the HECT N-lobe (designated as “front”) and on the opposite face of the N-lobe near the α1-helix (designated as “back”). This helix has been implicated in controlling the flexibility of the C-lobe and is a target site for regulation of HECT domain autoubiquitylation (22).

To examine the role of the HECT–UEV interaction in the cell, we used a previously established reporter assay for Nedd4-2s–mediated rescue of HIV-1 pNL4-3-ΔPTAP particles from infected human (293T) cells. pNL4-3-ΔPTAP encodes a *gag* gene lacking the intact Tsg101 binding site (through substitution of LIRL). The Nedd4 isomer Nedd4-2s possesses a truncated C2 domain derived from alternative splicing (23) and was previously shown to bind HIV-1 Gag directly and to account for the residual titer of the ΔPTAP construct (18, 19).

Here, we provide evidence that the interaction of the Tsg101 UEV domain with the catalytic domain of Nedd4-1 plays a key role in production of infectious HIV-1 particles and identify critical determinants of the rescue within the UEV and HECT domains. The UEV regions in proximity to the HECT lie on the β-hairpin and in proximity to the vestigial active site. These regions bind Ub noncovalently (24, 25). As noted above, on the front surface of the HECT, regions in proximity to the UEV lie in the interlobe region near the canonical E2 binding site and noncovalent Ub exosite, and on the reverse surface, the UEV was found to associate with the α1-helix region. We then tested for functional significance by mutating sites in these HECT regions in an established HIV-1 reporter assay using the related Nedd4 isoform Nedd4-2s. Mutation of residues in a region encompassing the interlobe hinge and the N-lobe portion of the E2 binding site prevented rescue of infectious particles without major impacts on viral particle release efficiency, suggesting that the HECT and HECT-UEV interaction are involved in the post budding processes that drive viral infectivity. The results highlight a multisite region of the HECT that may be stabilized or conformationally modulated by interaction with the Tsg101 UEV domain and that could serve as a novel target for antiviral drug design.

## Results

### Tsg101 UEV domain and Nedd4-1 HECT domain interaction

**Figure 1:**
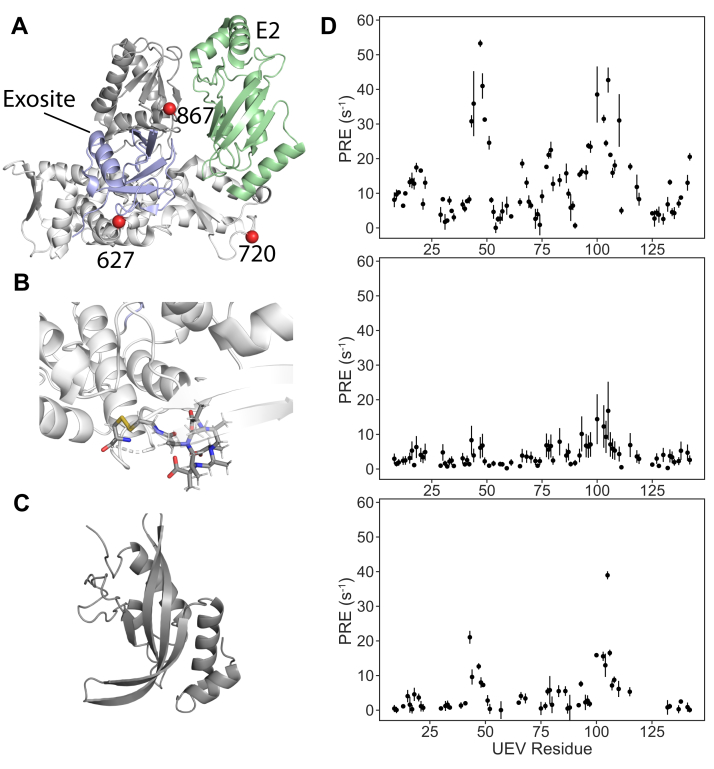
**The structural organization of the Nedd4-1 HECT domain, lanthanide tag, and Tsg101 UEV domain.***A*, front view of the Nedd4-1 HECT domain (PDB ID: 4BBN), with flexibly connected N- (*bottom*, *white*) and C- (*top*, *gray*) lobe organization. The E2 (*green*, from UbcH5B structure, PDB ID: 3JVZ) and the exosite (*blue*, from bound Ub in 4BBN) binding mode are also shown. The PRE restraints (*red spheres*) are located in the N-lobe at site 627 in the exosite and at site 720 below the canonical E2 binding site and in the C-lobe at the active site cysteine residue, C867. *B*, a representation of the lanthanide DOTA tags used in this work, highlighted at position 627. *C*, the Tsg101 UEV domain. *D*, PRE data collected for sites 627 (*top*) and 720 (*middle*) in the N-lobe and site 867 (*bottom*) in the C-lobe. HECT, homologous to E6AP carboxyl terminus; Nedd4-1, neural precursor cell-expressed developmentally downregulated 4-1; PRE, paramagnetic relaxation enhancement; Tsg101, tumor susceptibility gene 101; Ub, ubiquitin; UEV, ubiquitin E2 variant.

The UEV domain of Tsg101 resembles canonical E2 Ub-conjugating enzymes but is unable to catalyze Ub transfer because it lacks the active site cysteine that forms the transient thioester bond with the C terminus of Ub (26, 27). Moreover, it binds Ub but at novel sites that are not fully equivalent to that in canonical E2s (24, 25, 28). Still, given the similarities, we initially probed for evidence of a direct interaction between the Nedd4-1 HECT domain and the Tsg101 UEV domain at three sites (Fig. 1*A*, *red spheres*) in the vicinity of the E2 binding location on the HECT domain. Site 867 is the location of the catalytic cysteine in the C-lobe, while site 627 is in the noncovalent Ub binding exosite of the N-lobe (Fig. 1*A*, *blue*) and site 720 is situated in a proximal β-hairpin. When visualized on the structure of Nedd4-1 (PDB ID: 4BBN) having a “T” shaped interlobe configuration akin to the E2-bound structure of Nedd4L (PDB ID: 3JVZ), these three locations triangulate the E2 interaction region (Fig. 1*A*, *green*) (29, 30). We collected NMR PRE data for each location, using Nedd4-1 HECT labeled with a Gd-DOTA tag (Fig. 1*B*), and ^15^N labeled Tsg101 UEV domain (PDB ID: 4YC1, Fig. 1*C*). Data for each site are shown in Fig. 1*D*, with the largest magnitudes seen for site 627, followed by site 867 and finally site 720. The profiles of all three datasets are similar with significant enhancements seen on the UEV side in the V43-S48 region and in an area flanking the vestigial active site (T99-H115). The former is part of an unique extended β-hairpin element in the UEV (relative to canonical E2) that links β-strands 1 and 2. The latter region lies within the central active site region where structural similarity to canonical E2 is greatest (aa53–138) (24).

The data suggested the potential for interaction of the UEV domain with the HECT domain in and around the canonical E2-binding site and the Ub exosite, and we next sought to create representative models of such an interaction using the software package Xplor-NIH. Ensemble inputs were generated through *in silico* labeling of the HECT domain with the lanthanide tag at the relevant PRE sites, and then placing a single Tsg101 UEV domain 5 nm away from the HECT domain. Random rotation and translation of the UEV, followed by rigid body docking of the UEV domain to a fixed HECT domain, allowed an interaction of n UEV domains with a single HECT domain to be emulated with an ensemble of multiple HECT-UEV copies. In addition to the PRE data and the standard Xplor bond, angle, improper, and repel energy terms, previous NMR driven modeling of encounter complexes based on PRE or PCS restraints utilized a radius of gyration term to drive the initial docking between domains (31, 32). Adding this term allowed us to shift the representation of the PRE restraints in Xplor-NIH to the “correlation” mode, where the distance dependence is eased in favor of increased convergence toward correlation of the observed and calculated values.

### Modeling the HECT-UEV interaction around the E2 binding site and the Ub exosite

**Figure 2:**
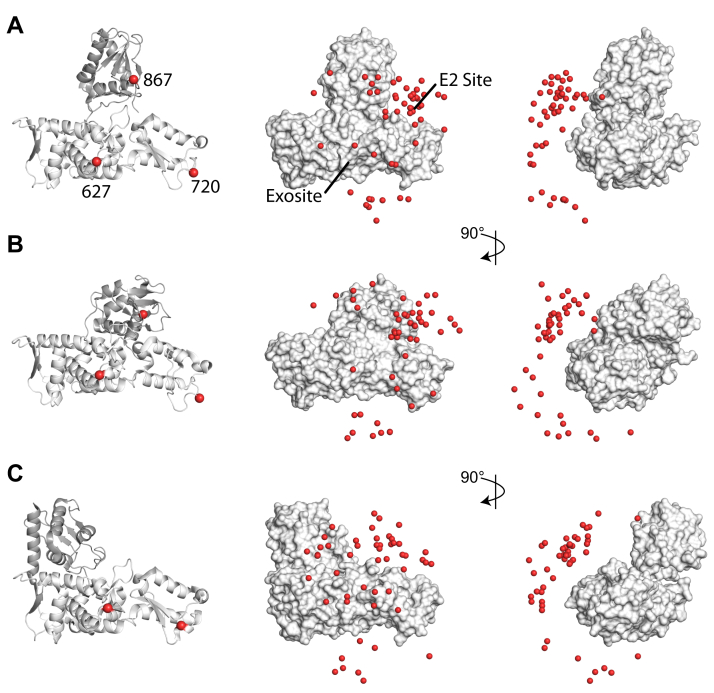
**Representative UEV positions for the top 10% of docked 5-member ensembles to varied HECT starting structures.***A*, cartoon view of the PDB ID: 4BBN structure (*left*) with PRE restraint sites 627 and 720 in the N-lobe and 867 in the C-lobe. The top 10% of docked UEV positions are then visualized onto the HECT structure in center of mass (*spheres*) representation for both front (*middle*) and side (*right*) views. *B*, same as in (*A*) for the 4BE8 starting structure. *C*, same as in (*A*) but for the 5C7J starting structure. For this case, the 867 restraint points away from the 627 and 720 sites and was omitted from the docking. In each case, jointly satisfying the PRE restraints places UEV centers primarily near the canonical E2 site, as expected given the similarity of the Tsg101 UEV domain to E2 enzymes, that includes the interlobe region near the exosite. HECT, homologous to E6AP carboxyl terminus; Nedd4-1, neural precursor cell-expressed developmentally downregulated 4-1; PRE, paramagnetic relaxation enhancement; Tsg101, tumor susceptibility gene 101; Ub, ubiquitin; UEV, ubiquitin E2 variant.

We first screened the results of docking an increasing number of UEV domains to a 4BBN conformation HECT domain (Fig. S1*A*). The site 867 restraints in the C-lobe were almost immediately satisfied, and only two UEV copies were needed to achieve a mean correlation for the top 10% of ensembles of > 0.9. For site 627, five UEV copies were sufficient, and for the restraints from site 720, ten UEVs were required. Figure 2*A* shows the results visually for the case where five UEV domains are docked to the HECT domain, with the centers of mass (*spheres*) shown for the top 10% of UEV domains. In the front view (Fig. 2*A*, *middle*), it is clear that with the current PRE restraints, the UEV domains clustered both in the Ub exosite and in the interlobe region around the canonical E2 site. From the side, the interaction surface extended from the C-lobe, down to below the N-lobe, again with the most density around the interlobe region and E2 binding site.

The C-lobe of the HECT domain is thought to have significant conformational flexibility that may pertain to its E3 ligase function, and structures of HECT ligases have been determined in a variety of N- to C-lobe orientations. To partially encode this flexibility into the modeling, we added two additional starting structures. In addition to the “T” shaped conformation seen in PDB ID: 4BBN (Fig. 2*A*, *left*), the structure of Nedd4-1 with the A889F mutation (PDB ID: 4BE8, Fig. 2*B*, *left*) results in a “tilted” lobe conformation as in the structures of the Nedd4 isoform WWP1, including PDB ID: 1ND7 (29, 33). In both cases, the C-lobe catalytic cysteine (site 867) is oriented toward the E2 site and exosite, forming a single apparent binding surface with sites 627 and 720 in the N-lobe. The correlations of the three restraints for the 5 and 10 UEV conditions are equivalent to the 4BBN structure (Fig. S1*B*, *left*), and a visual representation of the results for five UEVs (Fig. 2*B*, *middle and right*) again features clustering of the UEV domains into the interlobe region near the E2 binding site reaching toward the Ub exosite.

Alternatively, in the “L” shaped HECT structures such as PDB ID: 5C7J (Fig. 2*C*, *left*), the C-lobe is twisted such that the catalytic cysteine points away from the E2 and noncovalent Ub-binding sites and instead points toward the α1-helix element on the opposite surface of the N-lobe (34). This moves site 867 to an orientation opposite that of sites 627 and 720. Repeating the docking using the 5C7J structure and only the 627 and 720 restraints showed little improvement in correlation from the removal of site 867 (Fig. S1*B*, *middle*), with the primary change in the visual results with five UEV domains (Fig. 2*C*) being an increase in the apparent spread of the ensembles. For the lobe conformation seen in 5C7J, the interaction at site 867 would indicate either the presence of a secondary binding surface on the opposite face of the N-lobe or the extension of the current binding surface to that face.

### Modeling the HECT-UEV interaction near the α1-helix

**Figure 3:**
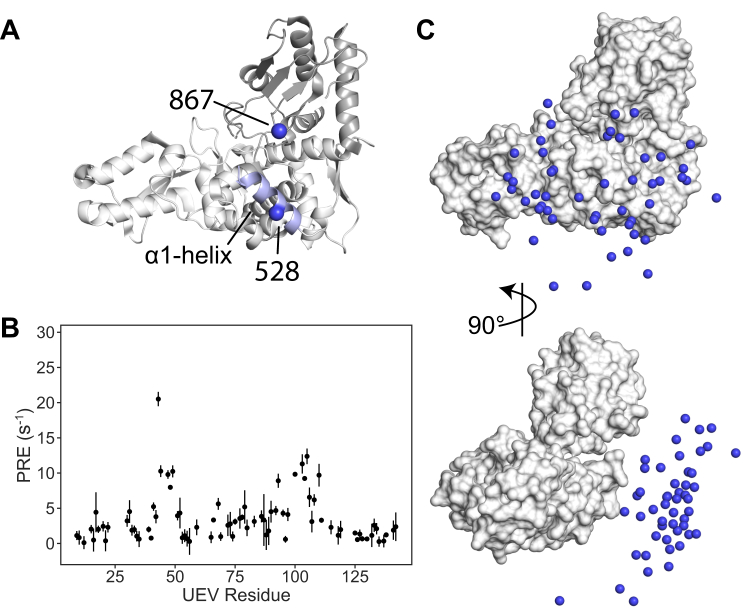
**Representative UEV positions for the top 10% of docked 5-member ensembles to the PDB ID:****5C7J****HECT structure focused on the α1-helix region.***A*, cartoon view of the PDB ID: 5C7J structure, shown as a 180-degree rotation relative to Fig. 2, with PRE restraints at sites 867 in the C-lobe and site 528 in the α1-helix. *B*, PRE data obtained for HECT labeled at site 528. *C*, the *top* 10% of docked UEV positions are then visualized onto the HECT structure in center of mass (*spheres*) representation for both front (*top*) and side (*bottom*) views. HECT, homologous to E6AP carboxyl terminus; UEV, ubiquitin E2 variant; PRE, paramagnetic relaxation enhancement.

To distinguish the presence of a second binding surface on the opposite face of the N-lobe from an extension of the front to the back surface, we added a PRE restraint at site 528 (Fig. 3*A*) in the α1-helix. The resulting PRE data (Fig. 3*B*) again showed a HECT-UEV interaction, with the same preferences for the β-hairpin and the region proximal to the vestigial active site as in the other datasets. Modeling this interaction showed good correlation for both five and 10 membered ensembles Fig. S1*B*, right). A representation is shown for the five UEV copies case in Fig. 3*C*, although with only two restraints the interaction appears to cover most of the surface of the HECT domain. Still, this points to either a secondary interaction near the α1-helix or to a contiguous interaction surface connecting the exosite, E2 site, and α1-helix region.

### Increasing the number of UEVs in the HECT-UEV model and pseudo-contact shifts reveal encounter-like nature of HECT-UEV interaction

Exploring the results for 10 UEVs per HECT visually, which was required for a site 720 correlation > 0.9, produced a greater spread of UEV placements (Fig. S2) and emphasized the potential for the E2, exosite, and α1-helix interactions to represent one binding surface. Future work with additional PRE sites in the HECT domain will be necessary to determine the simultaneity and overlap of these binding regions. Additionally, to examine whether there was orientational dependence to the interaction, we collected PCS data for site 627 (Fig. S3). While the shape of the data again appeared to show preference for the β-hairpin, the region proximal to the vestigial active site, and the N terminus, the magnitude of the data was very small*, i.e*., below the cutoff used in a previous characterization of the adrenodoxin: cytochrome c complex (35). Thus, it appears that the HECT–UEV complex is encounter-like and has a very weak orientational preference.

### Mutation of HECT sites within the Tsg101 interaction region impairs HIV-1 production

To obtain evidence that the HECT-UEV interaction plays a physiological role, we employed a reporter assay that employs Nedd4-2s to rescue the severe budding and infectivity impairment resulting from disruption of Tsg101 direct interaction with the HIV-1 structural precursor polyprotein Gag. The construct HIV-1 pNL4-3 ΔPTAP encodes a mutation in Gag that blocks infectious virus production. As noted above, Tsg101 participation is required for Nedd4 rescue, but direct binding of the protein to Gag is precluded by removal of the Tsg101 recognition site PTAP (17, 18, 19).

### Validation of the Nedd4-2s-mediated virus rescue reporter system

As shown in Fig. S4, WT Nedd4-2s rescued both release and production of infectious HIV-1, confirming previous findings using the reporter system. While previous studies reported an unspecified increase in NL4-3 release efficiency and as much as 40-fold for Gag ΔPTAP (Chung 2008), we observed 2- to 10-fold increases in NL4-3 budding efficiency (Fig. S4, *compare lanes 1–4 to lanes 25–28*) and 8-fold for a Gag variant encoding a disrupted PTAP motif (not shown). For infectivity, previous studies reported 15- to 40-fold increases while we observed 50- to 100-fold stimulation. As in previous studies, Nedd4L/Nedd4-2s overexpression did not alter cellular Gag protein levels, indicating that the titer increases were not simply due to elevated Gag expression. Finally, previous studies also found that deletion of the Nedd4L C2 conferred the ability to promote CA maturation inside the cell, compared to the full-length protein or deletion of the WW domain 2 (17). Nedd4-2s, whose C2 region is naturally truncated due to alternative splicing, conserves this property, and we found promotion of CA maturation to be dose-dependent for the WT Nedd4-2s protein (*c.f.*, Fig. S4).

It should be noted that previous studies attributed the strong positive impact of adventitious Nedd4-2s on cellular CA-spacer (CA-SP1) conversion to mature CA to reduction in budding delay (17). This speculation may be based on observations that disruption of viral L(ate) domains inevitably results in accumulation of immature particles with CA-SP1 that remain tethered to the plasma membrane, suggesting that coordination of virus egress, and maturation is facilitated by ubiquitination signaling. Supporting this possibility, we observed that WT Nedd4-2s promoted both events in a dose-dependent manner (*c.f.*, Fig. S4).

### Predictions from the model

Previous studies demonstrated that Nedd4-mediated rescue requires active enzyme and Tsg101 expression, although Tsg101 binding to Gag is not required (17, 18). The mechanism underlying rescue is unknown, and we hypothesized that the UEV-HECT interaction detected by NMR is involved. To test this, we determined the effect of substituting Ala for residues in the HECT domain in contact with the identified UEV β-hairpin and vestigial active site regions. Based on PRE and PCS data, sites were located in proximity to the hinge separating the N- and C-lobe subdomains, the N-lobe exosite, and the E2 binding region on the HECT domain front face. Mutants were tested for impact on viral particle release efficiency (a measure of the ESCRT rescue function) and for the ability to promote CA maturation in the cytoplasm. Previous studies observed that Nedd4-2s addition was uniquely effective in stimulation of CA-SP1 proteolysis to mature CA in the cytoplasm and suggested that this property was linked to its ability to relieve the budding delay associated with late domain impairment (17). CA maturation has previously been proposed as a correlate of viral infectivity (36), and so we also examined some sites directly for specific infectivity.

**Figure 4:**
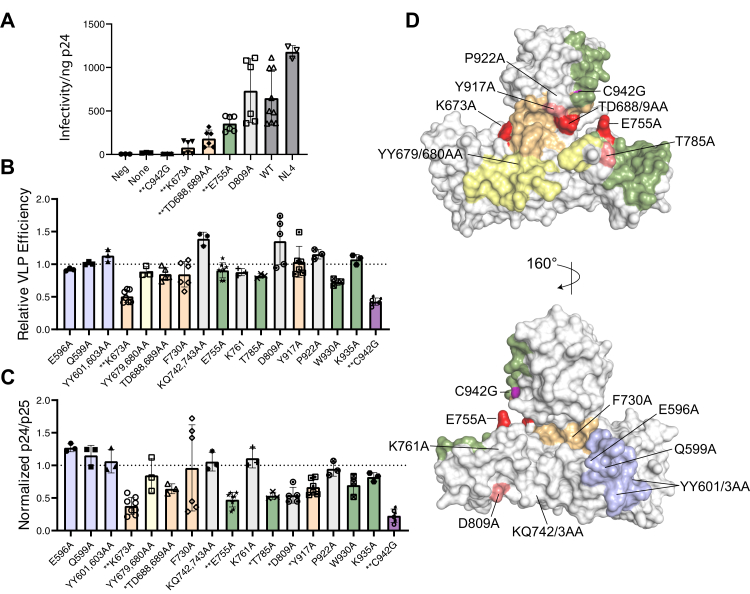
**Effect of HECT mutations on virus particle rescue.** Western blot signals from rescue assays were quantified relative to the Nedd4-2s WT samples run in parallel (as shown in the representative reporter assay in Fig. S4). *Panel A*, viral infectivity was measured by MAGI assay normalized to ng of CA protein detected by ELISA. (Negative is media only; None is no Nedd4-2s; C942G is catalytically inactive Nedd4-2s; WT is Nedd4-2s WT). Bars indicate ± one standard deviations from the mean. *Panel B*, viral release efficiency; *Panel C*, Nedd4-2s promotion of CA maturation. Results considered significantly different from WT are indicated (∗*p* < 0.05; or ∗∗*p* < 0.01; Student’s *t* test). *Panel D*, visualizing determinants of Ca maturation and viral infectivity onto the structure of Nedd4-2 (PDB ID: 3JVZ). Mutations employed in the efficiency, Ca maturation, and infectivity readouts are labeled on the Nedd4-2 surface. Infectivity determinants are colored red, while mutants exhibiting impaired Ca maturation are colored pink. For reference, functionally relevant HECT regions are also colored, including the noncovalent Ub exosite (*yellow*), the E2 binding site (*green*), the interlobe hinge region (*orange*), and the α1-helix (*blue*). Determinants of infectivity and reduced Ca maturation are primarily localized to the interlobe hinge region and the adjoining portion of the E2 binding site, implicating the HECT: UEV interaction in the conformational state of the HECT domain. Figure generated using pymol. The E2 binding site was approximated as residues within 5 Å of the UBCH5B domain in the 3JVZ structure of Nedd4-2, the exosite as residues within 5 Å of the noncovalent Ub after overlay of the corresponding Nedd4-1 structure (PDB ID: 4BBN) and the interlobe hinge region by finding N-lobe residues in the 3JVZ HECT within 5 Å of the C-lobe and vice versa. HECT, homologous to E6AP carboxyl terminus; UEV, ubiquitin E2 variant.

Starting with infectivity, severe impairments were seen for ala substitution of K673 (K673A), T688 in combination with D689 (TD688/689AA), and E755 (E755A) in the interlobe hinge and E2 binding regions of the HECT, respectively (Fig. 4, *panel A*). Notably, only K673A strongly reduced viral particle release efficiency (*panel* *B*) with most other mutants having WT level efficiency, indicating that although both budding (through effective ESCRT III recruitment) and infectivity are aspects of Nedd4-2s-mediated rescue, the HECT domain and HECT-UEV interaction primarily contribute to maturation and infectivity. Consistent with this, two mutants: K673A and E755A, showed both a strong reduction in infectivity and an inability to promote dose-dependent CA maturation (*panel C*). K673A lies in the interlobe hinge while E755A is part of the E2 binding site in the structure of Nedd4-2 in complex with UbcH5 (PDB ID: 3JVZ). Still, in other lobe conformations, including the alternate tilted conformation (as in PDB ID: 4BE8) used in the modeling of the HECT-UEV interaction by NMR, E755A may form its own interlobe contacts (Fig. S6). The final infectivity determinant, TD688/689AA, did not have a strong impact on CA maturation, indicating that this region of the interlobe hinge may impact a different point in the post-budding events leading up to particle infectivity. Finally, three additional mutants Y917A in the interlobe hinge, E785A in the E2 site, and D809A were found to be determinants of CA maturation (Fig. 4*C*).

The results obtained from the Nedd4-2s reporter assay are summarized visually in Figure 4*D* onto the PDB ID: 3JVZ structure of Nedd4L. Determinants of infectivity (red) are localized in and around the interlobe hinge (orange), with K673A and TD688/689A being located in the N-lobe portion of the hinge region, while E755A straddles the hinge and the E2 binding site (green). Two determinants of CA maturation expand this picture, with Y917A in the C-lobe being proximal to the TD688/689AA pair, while E785A is located in the β-hairpin portion of the E2 binding site near E755A. Interestingly, while the HECT-UEV interaction identified by NMR included the noncovalent Ub binding exosite in the N-lobe (yellow), mutation of YY679/680 in this region had no significant impacts. These residues are the Nedd4-2s equivalents of YY604/605 which have previously been characterized as determinants of noncovalent Ub association at the exosite, implying that viral rescue by Nedd4-2s and the HECT-UEV interaction are not dependent on Ub binding in this region (29).

Despite a propensity for HECT-UEV contact at the α1-helix, there was minimal impact from mutating sites on the back face of HECT in the Nedd4-2s reporter assay, with only D809A showing a CA maturation phenotype. This site was a potential location of HECT-UEV contact based on the NMR driven modeling but is not in a region of known functional significance on the HECT and did not show a concomitant reduction in specific infectivity. The α1-helix is known to be involved in regulation of HECT E3 ligases, but a recent model of HECT domain regulation implicates both the α1-helix and C2 domain of Nedd4 family E3 ligases as structural determinants of HECT regulation. Thus, the lack of phenotypes in this region may reflect upon the presence of a truncated C2 domain in the reporter assay.

Overall, the results of the Nedd4-2s reporter assay suggest that the interlobe hinge extending into the N-terminal region of the E2 binding controls viral infectivity, suggesting that multiple regions of the HECT domain function together in concert. The infectivity phenotype also correlated to CA maturation, as evidenced by the results for K673A and E755A. This coupling and the observation that other than K673A, mutants with reduced infectivity or impaired processing did not significantly alter particle release efficiency, suggests that the HECT domain of Nedd4-2s and the HECT-UEV interaction function differentially in the critical postbudding events required for infectious particle formation.

**Figure 5:**
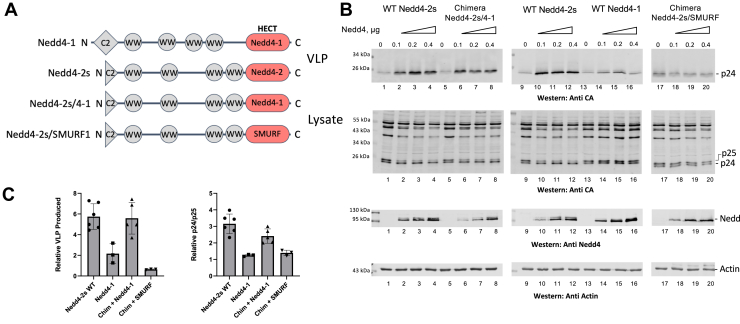
**The HECT domains of Nedd4-1 and Nedd4-2s are equivalent in their rescue of pNL4-3-LIRL (ΔPTAP).***A*, domain architecture of the Nedd4 constructs used to test HECT domain equivalency. WT sequences of Nedd4-1 (*top row*) and Nedd4-2s (*second row*) were compared to chimeras constructed with the HECT domain (*pink*) of Nedd4-1(*third row*) or SMURF1 (*bottom row*) substituted for the HECT domain of Nedd4-2s. *B*, 293T cells were transfected with pNL4-3-LIRL alone or with the chimeras, Nedd4-2s WT, or Nedd4-1 WT. Cell lysates and VLP were analyzed by Western blot and probed as indicated. *C*, the Nedd4 constructs were compared for their ability to rescue pNL4-3-LIRL as determined by the levels of VLP and p24/p25 (lysate) detected on the Western blots. There is no statistical difference between the impact of Nedd4-2s WT and the chimeric Nedd4-2s + Nedd4-1 HECT; there are significant differences between Nedd4-2s WT *versus* Nedd4-1 WT (*p* < 0.01) and Nedd4-2s WT *versus* the chimera Nedd4-2s + SMURF1 HECT (*p* < 0.001) as determined by Dunnett’s multicomparison test. HECT, homologous to E6AP carboxyl terminus.

As noted above, our structural studies employing the HECT domain of Nedd4-1 identified the UEV β-hairpin and vestigial active site as the primary regions in contact with the HECT domain. As previously reported, these regions of the UEV bind mono-Ub and K63-linked Ub noncovalently (24, 25). Previous studies showed that certain isolated HECT domains capable of synthesizing K63-linked Ub chains promoted WT-level rescue when fused to the residual C2 domain of Nedd4-2s, while others did not. We hypothesized that the basis of this rescue is HECT recognition of Ub-interacting motifs in E2 and E2-like proteins. Figure 5 shows that the HECT domain of Nedd4-1 was capable of rescue at the WT Nedd4-2s level when substituted for the HECT domain of Nedd4-2s. In contrast, the HECT domain of SMURF was not, even though expressed at comparable levels. Alignment of Nedd4 family members shows that the ability to effectively substitute correlates directly to conservation of residues K673, T688, and D689 in Nedd4-1 and Nedd4L but not in SMURF (Fig. S5). Thus, we conclude that HECT domain recognition of K63-linked di-Ub binding determinants in the UEV of Tsg101 is critical for its function in promoting HIV-1 egress, maturation, and infectivity.

## Discussion

In addition to providing intramolecular information on protein structure, PRE and PCS restraints have the capacity to provide intermolecular information on the state of protein-protein complexes. When coupled with recent advances in the use of lanthanide complexed DOTA cage tags to increase both sensitivity and the observable distance range, this methodology can probe even weakly interacting complexes, which have previously been accessible only to other techniques. Encounter-like complexes identified *via* paramagnetic NMR include the N-terminal domain of enzyme I and the histidine-containing phosphocarrier complex, an association of two major bacterial signaling proteins; the interaction of cytochrome *c* with adrenodoxin, or the DR: KE dimer of the PB1 domain of p62, which is involved in several signaling cascades (32, 35, 37, 38, 39, 40). Here, we report the formation of a weak complex between isolated Nedd4-1 HECT and Tsg101 UEV domains that involves several regions on the HECT domain surface. Since HECT domains are highly conserved across the Nedd4 isoforms, we anticipate the findings could be representative of many catalytic domains in this enzyme family. It will be of interest to determine whether the UEV plays a similar role in regulating the activity of other members for which there are feasible reporter assays.

The UEV domain of Tsg101 is highly similar to E2 ligases, albeit without the active site cysteine (28). The PRE data obtained at all sites show a weak preference for the β-hairpin region from residues 40 to 50 of the UEV domain, and crucially, for a region between T99 and H115 that lies proximal to the vestigial E2 active site. This suggests that the UEV domain may sometimes bind in a mode analogous to an E2. Binding near the E2 site could provide a regulatory mechanism through competitive inhibition of the E2 ligase. Interestingly, however, the HECT-UEV interaction identified by NMR appears to extend beyond the expected E2-like mode to include the interlobe hinge region, the Ub exosite in the N-lobe and the α1-helix region on the opposite surface. While we cannot currently distinguish whether these might constitute separate events or be parts of a contiguous binding surface, binding of the UEV domain in these accessory locations could block Ub access, reducing HECT processivity. Consistent with this, work by Herrador *et al*. determined that the Vps23 UEV domain, acting *in trans* with both the E3 ligase Rsp5 and a substrate conjugated Ub, blocked further ubiquitylation of the substrate (20).

Binding to the Ub exosite has been suggested to play a role in substrate selection and recruitment (41), and thus, Tsg101 binding at the exosite could be reflective of Tsg101 as a HECT substrate. Similarly, on the back face of the HECT domain, the α1-helix has been shown to be an autoubiquitylation site (42). Binding of the Tsg101 UEV domain to HECT autoubiquitylated at the α1-helix may then facilitate Ub attachment to Tsg101 *via* the E3-isopeptide model of ubiquitylation (43) or vice-versa. These regions have also been implicated in the regulation of the HECT domain. In the front, UEV interaction at the E2-binding site and the exosite could switch activity from a processive to a distributive mechanism, curtailing Ub chain length, as previously demonstrated for mutations or small molecule binding in these regions (44). UEV interaction at both the exosite and the α1-helix could alter the autoinhibition of the HECT domain, as proposed in the “headset” autoregulatory model (45). For Nedd4-1 and -2, this would involve interference by the UEV domain with binding of the C2 domain at the exosite and/or the WW1 domain near the α1-helix (45). Alternatively, the α1-helix of the yeast E3 ligase Rsp5 in its native conformation disrupts oligomerization, while attachment of a covalent Ub analog to the α1-helix abrogates this effect (42). The authors proposed that the autoubiquitinylated α1-helix may exchange from the back face of the HECT domain to the front, allowing the Ub moiety to interact with the exosite, potentially altering the HECT conformation and oligomeric state of the protein (42). The Tsg101 interaction could then also serve to promote or hinder this transition.

To probe these possible models for the HECT–UEV interaction, we mutated sites near the Nedd4-1 HECT PRE probe sites in the related isoform Nedd4-2s. Unlike Nedd4-1, Nedd4-2s has a truncated C2 domain that allows it to directly bind the HIV-1 Gag protein and promote viral egress. We were thus able to use a previously characterized reporter assay to examine the impact of Nedd4-2s HECT domain mutants within the HECT-UEV interaction region on viral Gag protein processing, viral egress, and virus specific infectivity (17, 18, 19). Surprisingly, mutations in the exosite and α1-helix regions showed no significant phenotypes in the reporter assay. While this suggests that noncovalent Ub binding at the exosite is not critical to viral rescue by Nedd4-2s or to the HECT: UEV interaction, we cannot rule out the importance of the α1-helix. As noted, the proposed model of structural regulation for Nedd4-1 and Nedd4-2 involves backbinding of a WW domain to the α1-helix on the back face of the HECT and of the C2 domain on the front face. The truncated C2 of Nedd4-2s in our reporter assay, while advantageous in its ability to recruit the HIV-1 Gag protein, may abrogate this regulatory binding and limit the observed impact of the α1-helix element (45).

In contrast, dramatic phenotypes were seen for mutations near the cleft between the N- and C-lobes of the HECT domain. Indeed, the mutational results highlight residues that lie in the interlobe region of the Nedd4-2s HECT domain and also in the center of the Nedd4-1 HECT-UEV interaction triangulated by our front face PRE sites. The impact in all three assays was most apparent for K673A, which lies on one side of the interlobe cleft. Two additional mutants, TD688/689AA in the interlobe region and E755A that straddles the interlobe region and the E2 binding site, strongly reduced infectivity but displayed weaker (E755A) or no (TD688/689AA) release efficiency defect compared to K673A. Additionally, both K673A and E755A also displayed strongly impaired ability to promote CA maturation. This range of observed phenotypes may stem from the conformational flexibility of the HECT, which is thought to regulate HECT function (33). Consistent with this, two mutants displaying specific infectivity defects noted above (K673A and TD688/689AA) and one with a CA maturation defect (Y917A) have likely interlobe contacts in the T shaped Nedd4L crystal structure (PDB ID: 3JVZ). The proximal E755A mutation, between the interlobe site and E2 binding site, does not appear to contact the C-lobe in this conformation, but introducing even a minimal representation of conformational heterogeneity by way of a morph to the tilted conformation seen in the 4BE8 structure of Nedd4-1 reveals that this location also likely contacts the C-lobe (Fig. S6). Within this interlobe region, only the F730A mutation, which contacts only the interlobe linker and is likely involved in more transient associations, failed to produce CA maturation or infectivity phenotypes.

These results suggest that stabilization or maintenance of HECT conformation is critical to our observed CA maturation and infectivity defects. On this basis, we propose that the Tsg101 interaction is involved in alteration of relative interlobe conformation, affecting HECT domain enzymatic activity pertinent to the rescue functions. We thus propose that the HECT–UEV interaction may modulate the conformational state of the HECT domain either in a regulatory fashion to alter HECT processivity or in the context of Tsg101 as a HECT substrate.

In summary, the results presented here provide support for a novel protein–protein interaction involving the UEV domain of Tsg101 and the HECT domain of Nedd4-1 and its closely related isomers (Nedd4-2s and Nedd4-2 (Nedd4L). The encounter-like complex described here may be a transient state where one or more of the interfaces modeled precede a final, low energy state of the HECT-UEV interaction, or there may be one or more additional partner proteins *in vivo*. The solution NMR results with Nedd4-1 HECT are consistent with a broad interaction involving the canonical E2 binding site together with the noncovalent Ub exosite and the α1-helix region. Mutations in the E2-binding site and exosite of the related Nedd4-2s isoform tracked with an HIV-1 reporter assay revealed a narrower region of impact, where defects in CA maturation and specific infectivity are limited to the interlobe region. Together, our results suggest that the HECT-UEV interaction may play a role in the conformational landscape of the HECT protein, where the HECT-UEV interaction may either perturb the conformation of the HECT or stabilize it in an advantageous conformation. The results also provide novel insight into the manner in which Tsg101, a UEV protein, participates with the Ub E3 ligase Nedd4 in rescue of virus production. These studies could contribute to identification of targets in the enzyme useful for anti-viral drug design.

## Experimental procedures

### Production of Nedd4-1 HECT and Tsg101 UEV

All HECT domain constructs were created based on the human *Nedd4-1* (hNedd4-1) sequence. An N-terminal His_6_-tagged *hNedd4-1* HECT domain expression plasmid was engineered using the In-Fusion (Takara Bio) recombinational cloning approach. The *hNedd4-1* HECT domain coding sequence (corresponding to amino acid sequence 520–900) from the pCI-neo.mCherry-Nedd4 vector (46) was seamlessly inserted in-frame downstream to the TEV cleavage site already present in the pET-28b destination vector. The sets of primers, used to synthesize the PCR products corresponding to the insert and the destination vector, were designed using the SnapGene In-Fusion cloning tool (GSL Biotech) and ordered from Eurofins (Eurofins Genomics). The ligation-independent cloning was carried out following Takara’s recommendation. The single cysteine—C627 or C867—HECT domain plasmids as well as the null cysteine version—C627S, C778S, C867S—were prepared by nucleotide substitutions using the Q5 Site-Directed Mutagenesis Kit (New England Biolabs), following the manufacturer recommendation, and a pair of custom primers per mutation designed with the web-based NEBaseChanger and synthesized by Eurofins. The N-terminal His_6_-tagged N-lobe HECT domain (corresponding to amino acid sequence 520–780) was produced by introducing a point-nonsense mutation at residue G781 in the null cysteine N-terminal His_6_-tagged *hNedd4-1* HECT domain. Two overlapping primers containing an Opal codon were designed with the QuikChange Primer Design Program (Agilent) and synthesized by Eurofins; the QuikChange II XL Site-Directed Mutagenesis Kit was used following the manufacturer recommendation. Similarly, two sets of primers were designed to re-engineer two single cysteine mutants of the N-lobe HECT domain, namely F528C and S720C. The integrity of all the coding sequences was verified (Psomagen).

Rosetta 2 (DE3) pLysS competent cells (MilliporeSigma), transformed with the N-terminal His_6_-tagged *hNedd4-1* HECT domain or one of the N-terminal His_6_-tagged N-lobe HECT domain single cysteine constructs, were grown overnight at 37 °C in 1 L of Luria-Bertani broth (MP Biomedicals) containing 50 μg/ml kanamycin (Sigma-Aldrich) and 34 μg/ml chloramphenicol (Sigma-Aldrich). After dilution with 1 L of corresponding media containing the antibiotics, the cells were grown at 37 °C for 2 h before induction with 1 mM IPTG (EMD Millipore) for 20 h at 18 °C. The cell pellets, harvested at 6000 rpm for 20 min at 15 °C, were resuspended in 100 mM Tris·HCl pH 7.4 buffer, containing 500 mM NaCl, 0.5 mM TCEP, 10% (v/v) glycerol, and one cOmplete EDTA-free Protease Inhibitor Cocktail tablet (MilliporeSigma) and disrupted by two passages through an M-110P Microfluidizer (Microfluidic, IDEX Corporation). Cell debris removal was carried out at 185,500×*g* for 60 min at 4 °C. A 5 M imidazole (Sigma-Aldrich) solution adjusted to pH 8.0 was added to the supernatants to a final concentration of 40 mM. The supernatants were loaded onto a 5 ml HisTrap HP column (Cytiva) equilibrated with 25 mM Tris·HCl pH 7.4 buffer containing 500 mM NaCl, 1 mM TCEP, and 20 mM imidazole. The proteins were eluted, using a 100 ml linear gradient to 500 mM imidazole in the same buffer, as single peaks at a maximum imidazole concentration of ∼300 mM. The protein-containing fractions considered pure by SDS-PAGE were pooled, and their concentrations were estimated using their measured A_280_ and their respective calculated molar extinction coefficient (http://web.expasy.org/protparam/). TEV protease cleavages, using 1:100 enzyme over protein at A_280_, were carried out in 10K MWCO Slide-A-Lyzer Dialysis Cassettes (ThermoFisher Scientific), at RT overnight with a concomitant buffer exchange to 25 mM Tris·HCl pH 7.4 buffer containing 500 mM NaCl and 1 mM TCEP. The His-tagged proteins—TEV and uncleaved HECT domains—were removed by filtering the dialysates through a 5 ml HisTrap HP column (Cytiva) equilibrated in 25 mM Tris·HCl pH 7.4 buffer containing 500 mM NaCl, 20 mM imidazole, and 1 mM TCEP. After SDS-PAGE analysis, the protein-containing fractions were pooled and buffer exchanged into 50 mM Tris·HCl pH 7.4 buffer, 500 mM NaCl, and 5% (v/v) glycerol by ultrafiltration using an Amicon, 3K MWCO (Millipore). The concentration of the samples was estimated using their *A*_280_, and the identity of the proteins was confirmed by MS analysis (Agilent 6224 ESI-TOF LC-MS). Wild-type Tsg101 UEV domain (residues 2–145) was expressed and purified as a ^15^N-labeled protein, as described previously (47).

### Samples for NMR studies

Natural abundance HECTs with single cysteine at residue 528, 627, 720, and 867 were tagged with diamagnetic Lu-DOTA-M8-SPy or paramagnetic Tm-DOTA-M8-Spy for pseudo contact shifts, or Gd-DOTA-M8-Spy for PRE measurements, as described previously (48). Briefly, the hNedd4-1 HECT domain and N-lobe domain constructs, reduced with 2 mM DTT (Sigma-Aldrich) for 60 min at room temperature in 50 mM Tris·HCl pH 7.4 buffer containing 500 mM NaCl and 5% (v/v) glycerol, were applied onto a Sephadex G-25 PD-10 desalting column (Cytiva) equilibrated in the same buffer. The eluted proteins were added to a three-fold molar excess solution of either Lu-, Tm-, or Gd-M8-DOTA-Spy. The reaction mixtures were incubated for 16 h at room temperature, and completion was assessed by LC-MS (Agilent 6224 ESI-TOF LC-MS). The excess reagents were removed by buffer exchange to 50 mM Tris·HCl pH 7.4 buffer containing 500 mM NaCl and 5% (v/v) by ultrafiltration using an Amicon, 3K MWCO (Millipore). Tagged HECT samples were added to ^15^N-Tsg101 and concentrated in an Amicon ultracentrifugal filter (3K MWCO) to 250 μl for NMR measurement in a Shigemi tube. Final concentration of the NMR samples was 125 μM of ^15^N-Tsg101 to 150 μM of HECT. All samples were in a buffer containing 50 mM Tris (pH 7.4), 500 mM NaCl, 5% glycerol, and 7% ^2^H_2_O.

### NMR spectroscopy

All NMR spectra were acquired at 300 K on Bruker 600 MHz spectrometer equipped with cryoprobe. Spectra were processed using NMRPipe (49) and analyzed using CCPN Analysis 2.5.0 (50). Assignment of NMR resonances of Tsg101 UEV domain was described elsewhere (47). Transverse PRE rates for backbone amide protons (H^N^-Γ_2_) were calculated as the difference in the transverse relaxation rates (R_2_) between the paramagnetic and diamagnetic samples (H^N^-Γ_2_ = R_2_^para^ – R_2_^dia^). Amide proton transverse relaxation rates (R_2_) for paramagnetic and diamagnetic samples were acquired using an interleaved ^15^N–HSQC-based pulse sequence with two time points (20-ms separation) and 128 scans as previously described (51). The same two-time points were used for both the diamagnetic and paramagnetic species to eliminate effects from homonuclear modulation. Pseudo contact shifts were calculated from the difference between the amide proton chemical shifts obtained from ^1^H-^15^N HSQC spectra of ^15^N-labeled UEV mixed with the paramagnetic Tm-DOTA-HECT and the one containing diamagnetic Lu-DOTA-HECT. As a control, the PRE experiment was repeated with ^15^N-labeled UEV mixed with Gd-M8-Spy in the presence of excess amount of reducing agent (1 mM TCEP) to ensure that no disulfide bind formation occur between the DOTA tag and Cys residues in UEV domain, for which we observed no PRE.

### Modeling

All structure calculations used Xplor-NIH, version 3.1. The initial structures of the NEDD4-1 HECT domain were taken from PDB IDs: 4BBN, 4BE8, and 5C7J. The native cysteine at site 627 was missing from the 4BBN and 4BE8 structures, and so residues 620 to 630 from 5C7J were first spliced into the 4BBN and 4BE8 starting structures. All structures were then in silico labeled with two copies of the M8-DOTA-SPy tag (CTSA in Xplor-NIH) at the 528, 627, 720, and 867 positions, as needed. The UEV domain structure was taken from PDB ID: 4YC1. Initial ensemble inputs were generated by positioning the UEV domain 4 to 5 nm away from either the 627 or 528 sites on the HECT N-lobe, for the two binding representations shown in Figs. 2 and 3, respectively.

Docking used the standard Xplor BOND, ANGL, IMPR, repel, and torsionDB energy terms. PRE restraints used the “SBMF” option, and the “correlation” mode PCS restraints used the rdcPot term with setUseDistance set to True. For all runs, backbone atoms were held rigid and sidechain atoms were mobile in both the HECT and UEV domains, with the exception of the CTSA tag DOTA cage and metal center atoms, which were grouped. The location of the HECT domain was then held fixed during docking, while the UEV domain was allowed to move as a rigid body.

For the main docking protocol, following randomization of sidechain torsions, an initial 100 step sidechain-only minimization was used to orient sidechains. Subsequently, the UEV domain was translated by a random vector within a 6 nm sphere about its starting position, and 200 steps of joint minimization with repel and radius of gyration terms were used to “dock” the UEV domain to the HECT. After, the UEV domains were rotated randomly about their center of mass 20 times, selecting for the configuration that gave the highest joint PRE correlation. This initial process of translation, docking, and rotational search was repeated 10 times, with the best configuration used in subsequent steps.

Following the initial docking search, 1500 steps of torsion-angle minimization with all potential terms were followed by an additional 1500 steps without the radius of gyration term, to fix residual clashes. Finally, 500 steps of cartesian minimization with all energy terms except the radius of gyration was perfomed with rigid backbone atoms and free sidechains, before final structures were written. For all runs, we used the best ensemble or statistics on the best 10% of ensembles, as written. All runs were a total of 100 written ensembles. The addition of simulated annealing was not found to significantly improve results over the use of Powell minimization.

Results were analyzed using the standard Xplor-NIH outputs and in-house python scripts. All visualizations were generated using pymol and plots were generated using the seaborn, matplotlib, or plotly python packages.

### Plasmids and reagents

HIV-1 pNL4-3ΔEnv was as previously described (52).The p6 Late domain of pNL4-3ΔEnv was altered by site-directed mutagenesis from PTAP coding sequence to LIRL. For the experiments described here, that construct is noted as pNL4-3 LIRL and is designated as PTAP(−). Nedd4L (Genbank, AAP75706.1) and Nedd4-2s (GenBank, AB007899.1) were kind gifts from F. Bouamr (NIAID). pCMV5B-Flag-Smurf1 WT was a gift from Jeff Wrana (Addgene plasmid # 11752) (53). Mutations in Nedd4L and Nedd4-2s were created using site-directed mutagenesis and confirmed by DNA sequencing. The numbering for amino acids targeted for mutagenesis is based on Nedd4L. The Nedd4-2s plus Nedd4-1 chimera was constructed using the oligo 5′-tggtccggct gtcccttactccagggattacaaaagaaagtatgagttcttccg-3′ to stich the two Nedd sequences together. The Nedd4-2s plus SMURF1 chimera was constructed using the oligo 5′-ttactggtccggctgtccctacgaaagagatctagtccagaa gctgaaagtcctca-3′ to stitch the sequences together.

### Assays

293T cells (ATCC CRL-3216) were transfected using Roche X-tremeGene transfection reagent (Sigma-Aldrich). At 24 h, cells were collected, washed, and lysed (50 mM Tris, pH 7.4, 137 mM NaCl, 1.5 mM MgCl2, 1 mM EDTA, 1% Triton X-100, Roche complete mini protease inhibitor cocktail) and centrifuged at 1000*g* for 15 min. Supernatants were added to sample buffer and examined by Western blotting. For analysis of the virus-like particles, media from the cells were filtered (0.45 micron) and then pelleted through a 20% sucrose cushion by centrifuging at 20,000*g*, 90 min. After centrifugation and washing, pellets were resuspended in sample buffer and examined by Western blotting. Primary antibodies were Rb anti-CA (54); mouse anti-actin (Sigma-Aldrich, A4700); and mouse anti-Nedd4L (Santa Cruz Biotechnology., sc 514,954). Secondary antibodies were goat anti-mouse IgG IRDye 680; goat anti-mouse IgG IRDye 800; and goat anti-rabbit IRDye800. An infrared-based imaging system (Odyssey, LI-COR Biotechnology) was used to measure signal and the band intensities calculated using the Li-Cor Odyssey software, version 2.1.15. For multinuclear activation of a galactosidase indicator infectivity assays, 293T cells were cotransfected with pNL4-3 and pHIV-1-IIIB Env and Nedd4-2s WT or mutants as indicated in the text. Lysates from transfected 293T cells were analyzed by Western blotting to check for expression of pNL4-3-ΔPTAP and Nedd4-2s. The filtered media from the transfections were analyzed for p24 levels using ELISA (Immunodiagnostics Inc), and equivalent amounts of p24 were used to infect HeLa CD4^+^ LTR-βgal cells. Infectivity was measured using multinuclear activation of a galactosidase indicator assay (55). GraphPad Prism 9 software (GraphPad Software) was used to analyze the data produced by the Western blot and infectivity assays.

## Data availability

All data are contained within the article and the supporting information, except for raw NMR spectra. They are available upon request to the corresponding author.

## Supporting information

This article contains supporting information.

## Conflict of interest

The authors declare that they have no conflicts of interest with the contents of this article.
