# Supplementary material for: HECT domain interaction with ubiquitin binding sites on Tsg101-UEV controls HIV-1 egress, maturation, and infectivity
Source: J Biol Chem. 2023 Jan 13;299(2):102901. doi: 10.1016/j.jbc.2023.102901 (PMC9944984; doi:10.1016/j.jbc.2023.102901)
Supplement: Supporting information [file mmc1.pdf]

# HECT Domain Interaction with Ubiquitin Binding Sites on Tsg101-UEV Control HIV-1 Egress, Maturation and Infectivity

**David A. Nyenhuis[1#], Rohith Rajasekaran[1#], Susan Watanabe[2#], Marie-Paule Strub[1], Mahfuz Khan[3], Michael Powell[3], Carol A. Carter[2*] and Nico Tjandra[1*]**

[1]Biochemistry and Biophysics Center, National Heart, Lung, and Blood Institute, National Institutes of Health, Bethesda, MD, 20892, USA. [2]Department of Microbiology & Immunology, Renaissance School of Medicine, Stony Brook University, Stony Brook, NY 11794-5222, USA. [3]Department of Microbiology & Immunology, Morehouse School of Medicine, Atlanta, GA 30310, USA.

Running Title: Tsg101 UEV and Nedd4 HECT Domain Interactions

# These authors contributed equally
*To whom correspondence should be addressed:
Carol Carter: carol.carter@stonybrook.edu; Tel. (631) 632-8801
Nico Tjandra: tjandran@nhlbi.nih.gov; Tel. (301) 402-3029

**Keywords:** Nedd4, Ubiquitin, Tsg101, HECT, UEV, NMR, viral budding, endocytic sorting

**Supplementary Material**

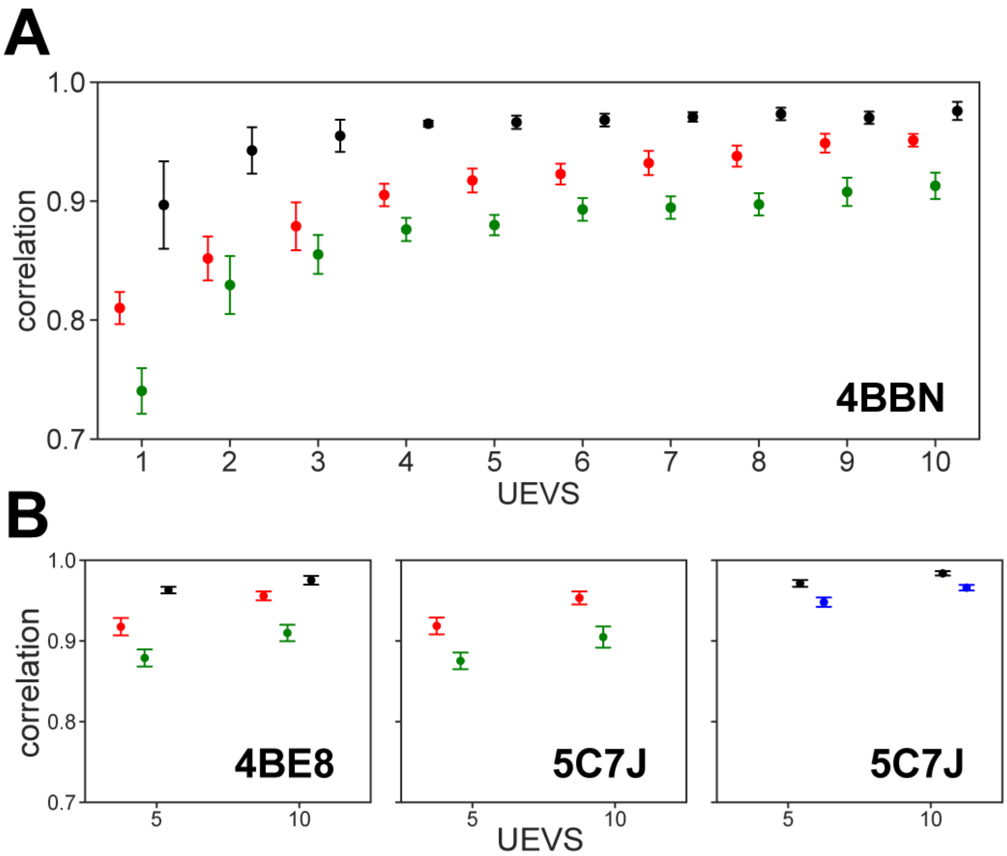


**Figure S1. PRE Restraint Correlation as a function of the number of UEV domains docked to a given HECT structure.** (A) Results for docking an increasing number of ensemble units, representing an increasing number of UEV domains, for the site 627, 720, and 867 PRE restraints to the PDB ID: 4BBN NEDD4-1 HECT structure. The mean correlation coefficients for the top 10% of ensembles are shown for sites 627, 720, and 867 in red, green, and black, respectively. Attaining correlations of greater than 0.9 for the N-lobe restraints requires 5 ensemble members for site 627 and 10 for site 720, while the C-lobe restraint at site 867 is almost immediately satisfied. (B, left) Comparitive correlation results for docking 5 or 10 ensemble members (UEV domains) to HECT for the 4BE8 (left) structure with restraints at sites 627, 720, and 867. (B, middle) The same for the 5C7J starting structure with restraints at sites 627 and 720. (B, right) Results for the 5C7J starting structure, with restraints at sites 528 in the $\alpha$-1 helix and site 867 in the C-lobe.

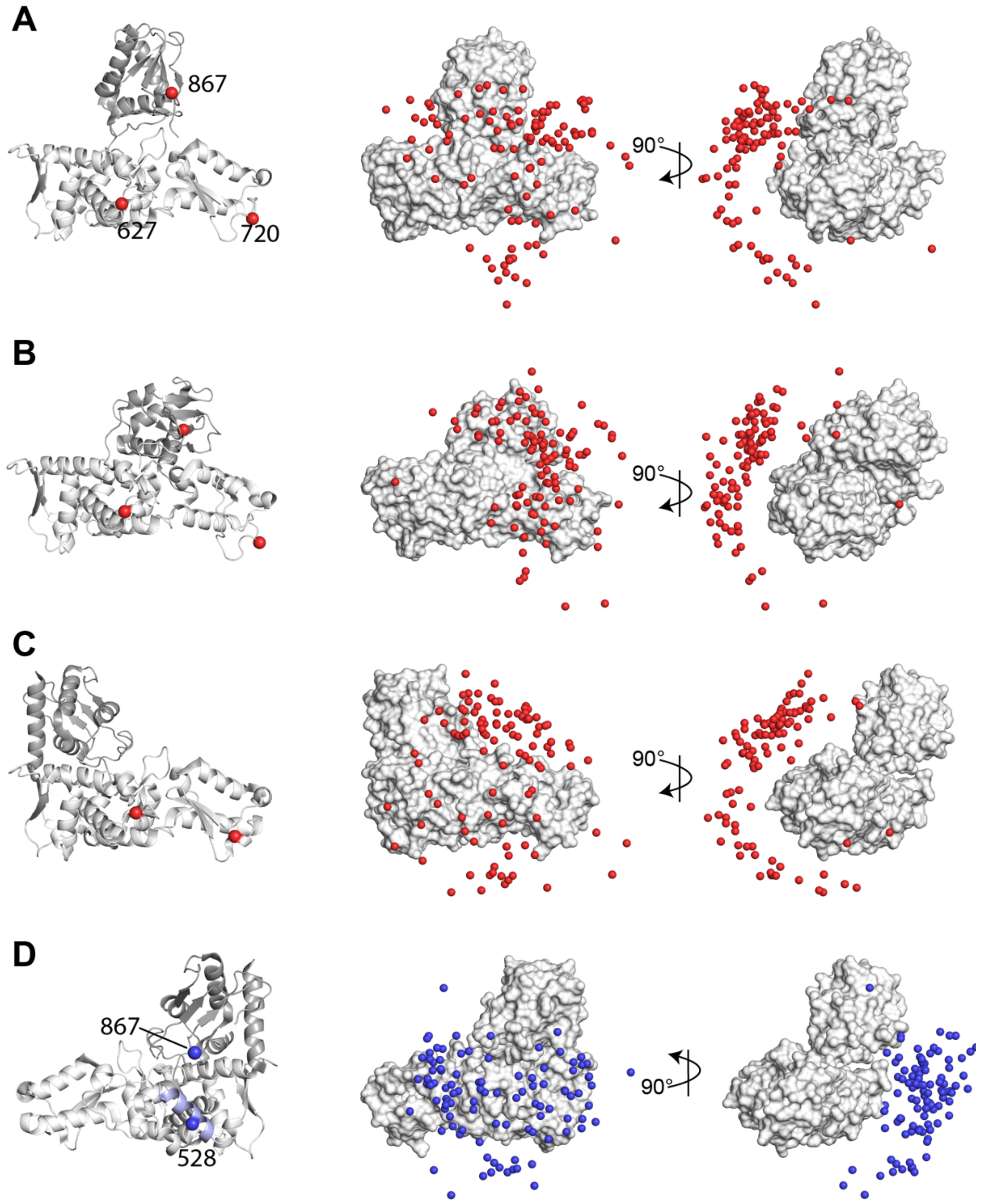


**Figure S2. Representative UEV positions for the top 10% of docked 10-member ensembles to varied HECT starting Structures.** (A) Cartoon view of the PDB ID: 4BBN structure (left) with PRE restraint sites 627 and 720 in the N-lobe, and 867 in the C-lobe. The top 10% of docked UEV positions are then visualized onto the HECT structure as centers of mass (spheres) for both front (middle) and side (right) views. (B) Same as in (A) for the 4BE8 starting structure. (C) Same as in (A) but for the 5C7J starting structure. For this case, the 867 restraint points away from the 627 and 720 sites and was omitted from the docking. (D) Results for the 5C7J, with the HECT reversed to evaluate docking of UEV domains to the back face with PRE data from sites 528 and 867.

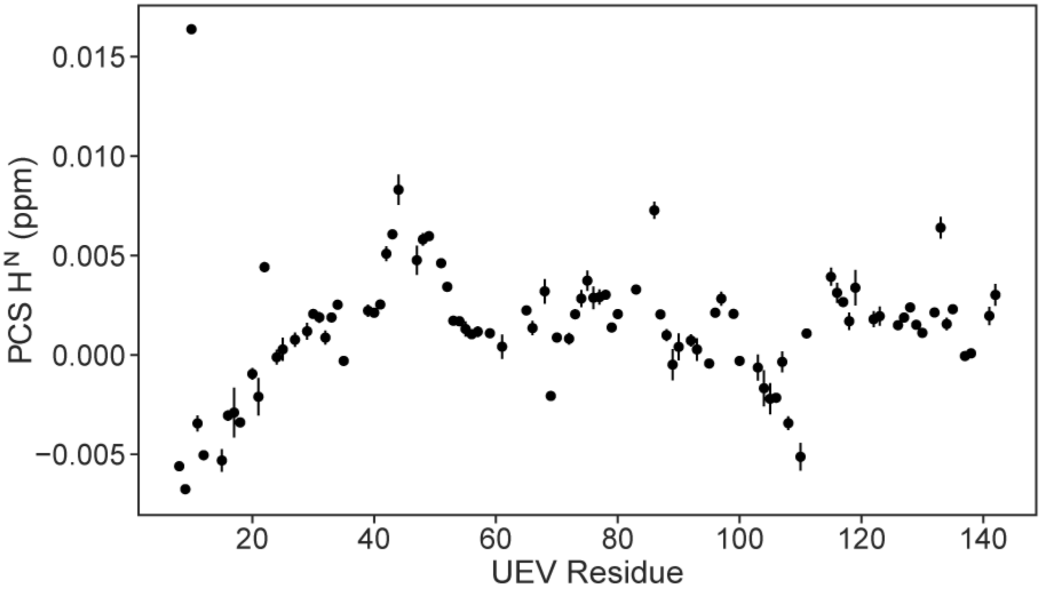


**Figure S3. $^1$H Pseudocontact shift data obtained for HECT labeled at site 627 with a Tm-DOTA tag and $^{15}$N labeled Tsg101 UEV domain.** Plotted data are the difference of amide proton shifts for spectra collected with Tm-DOTA tagged HECT relative to a Lu-DOTA tagged control. Some preference is observed in the dataset for the β-hairpin region (residues 40-50), but the magnitues are very small, indicating limited overall orientational preference.

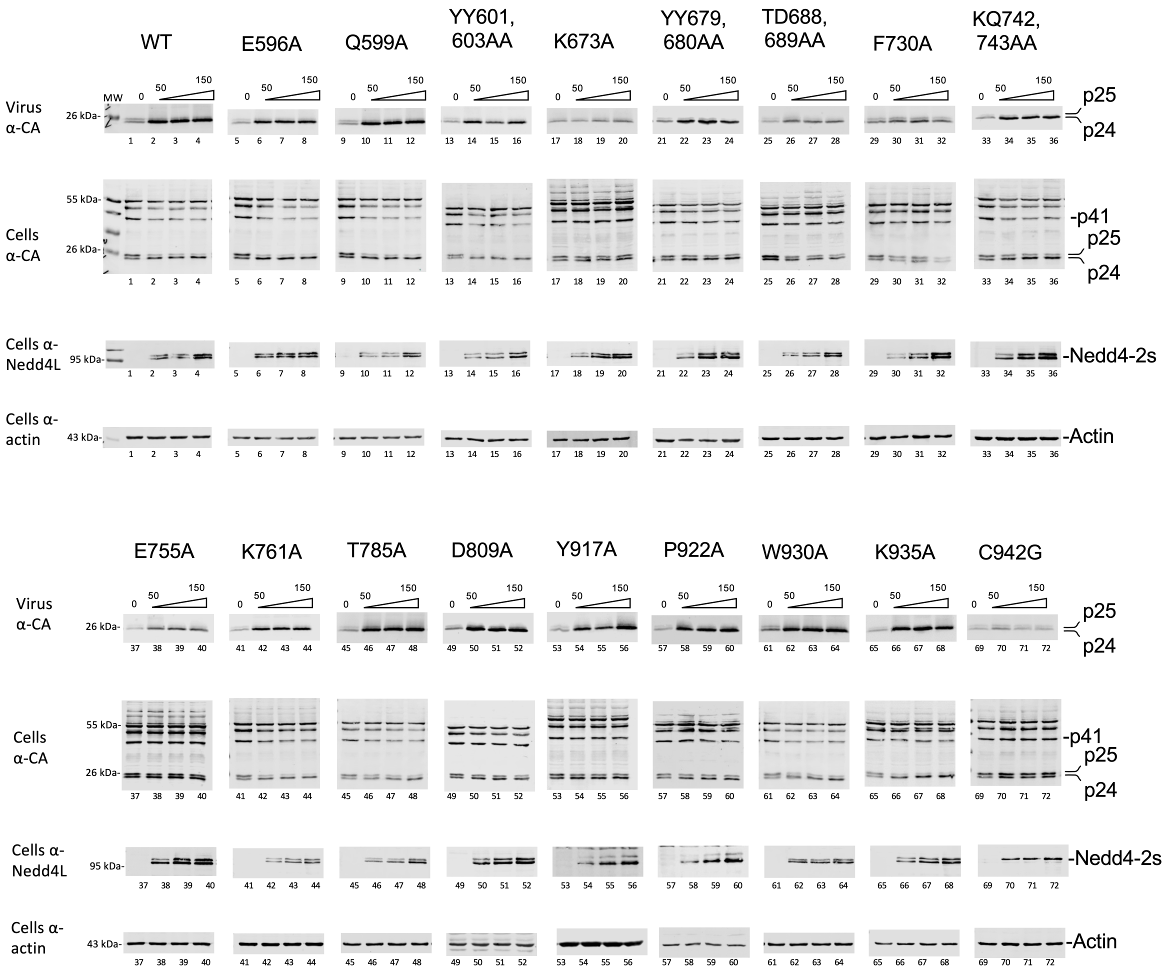


**Figure S4. HECT mutation effects on virus particle rescue and cytosolic stimulation of CA maturation.** Levels of virus particle release upon transfection alone or when co-transfected with increasing levels (50-150 ng) of the Nedd4-2s expression vector, WT or mutated, were analyzed by Western blotting and probed with the anti-capsid sera. Lysates of the transfected cells were analyzed by Western blotting and probed with antisera as indicated.

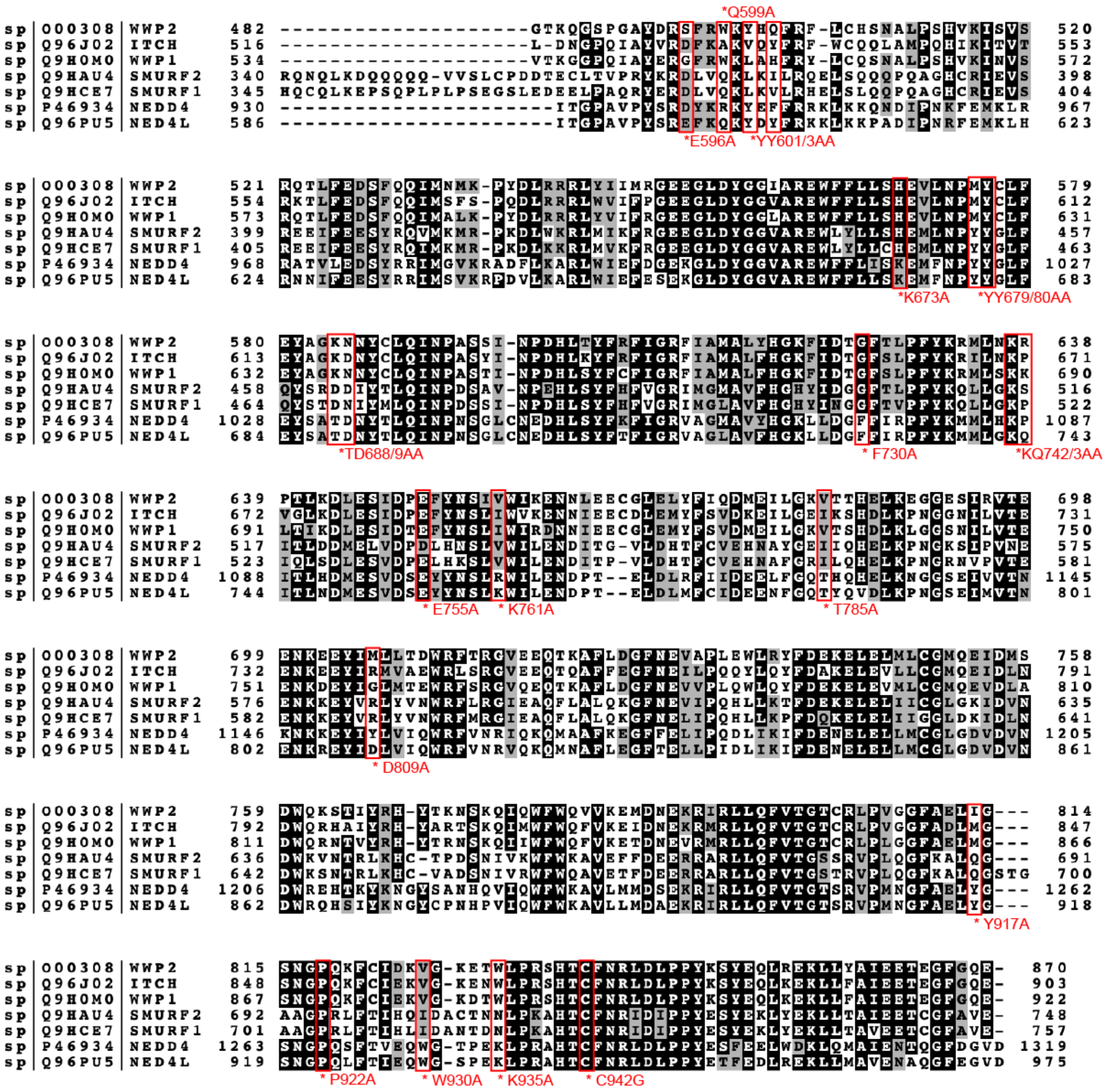


**Fig. S5. NEDD4 family alignments.** Only the HECT domain regions of each sequence are shown. Conserved residues are shaded black while similar residues are shaded gray. The mutations reported in this work are indicated in red. Eight of the mutated residues (K673, T688, Y917, W930, K935, T785, F730, Y601) are conserved only in the NEDD4-1 and -2 subgroup, three (Y680, D689, K742) are conserved in the related ITCH branch, and two are (E755, P922) are conserved broadly across HECTs. Three mutants (E596, Y603, K761) conserve charge and type between Nedd4-1 and Nedd4-2 and three (Q743, Q599, D809) are not conserved. The MSA was generated with clustal omega and visualized with the pyBoxShade package.

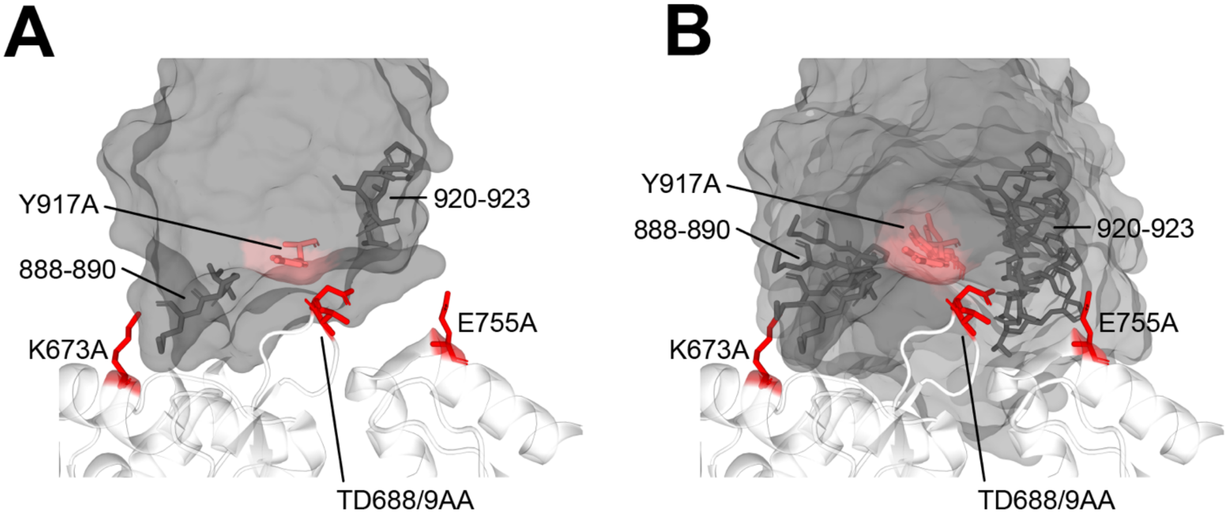


**Figure S6. Residues whose mutation adversely impact viral infectivity and CA maturation in a Nedd4-2s reporter assay are involved in likely interlobe contacts.** (A). A zoomed view of the interobe region of the Nedd4-2 HECT domain (PDB ID: 3JVZ) viewed from the front. Mutants with defects in infectivity (K673A, TD688,689AA, and E755A) are colored red while the Y917A mutant with defective CA maturation is shown in pink. Three of the mutants: K673, TD688,689AA, and Y917A are clearly positioned to make interlobe contacts within the T-shaped conformation captured by the 3JVZ structure. (B). A minimal example of how conformational heterogeneity might help explain the differential phenotypes seen for HECT mutants. The same mutants from (A) are shown, now displayed onto four conformations taken from a morph between the inverted T and tilted interlobe conformations seen for Nedd4-1 (PDB IDs: 4BBN and 4BE8), where in each case the C-lobe of 3JVZ was aligned to the corresponding position of the Nedd4-1 structural morph. As the conformation transitions from the T-shaped conformation towards the tilted orientation, potential contacts become visible for the E755A mutant, indicating that this site is likely also part of the interlobe hinge. In both panels, three residues in the C-lobe nearby K673A (residues 888-890) and E755A (residues 920-923) are shown for clarity.
